# Supplementary material for: A genome-scale CRISPR interference guide library enables comprehensive phenotypic profiling in yeast
Source: BMC Genomics. 2021 Mar 23;22:205. doi: 10.1186/s12864-021-07518-0 (PMC7986282; doi:10.1186/s12864-021-07518-0)
Supplement: Supplementary file 2 — Additional file 2: Figures S1 and S2. Detailed strategy for library construction and sequencing. [file 12864_2021_7518_MOESM2_ESM.pdf]

Cloning guide RNAs into pNTI661 sgRNA expression vector

```
Guide RNA expression vector:
pNTI661 …cgcggctgggaacgaaactctgggagctgcgattggGATCCTCGAAGCTttttagagctagaaatagcaagttaaaataaggctag…
          P(RPR1)                             BamHI   HindIII    sgRNA scaffold

Guide RNA amplification / assembly primers:
NM636 5'-ggctgggaacgaaactctgggagctgcgattggca
NM637 5'-gccttattttaacttgctatttctagctctaaaac

Guide RNA oligo pool:
pool  tctgggagctgcgattggcaNNNNNNNNNNNNNNNNNNNNgttttagagctagaaatagc
                P(RPR1)    guide RNA sequence    sgRNA scaffold

PCR amplification of guide RNA pool:
NM636       ggctgggaacgaaactctgggagctgcgattggca
pool                       tctgggagctgcgattggcaCATTTCATTACCCGCAGAGCgttttagagctagaaatagc
NM637 reverse complement                                           gttttagagctagaaatagcaagttaaaataaggc

PCR      5'-ggctgggaacgaaactctgggagctgcgattggcaCATTTCATTACCCGCAGAGCgttttagagctagaaatagcaagttaaaataaggc-3'

BamHI / HindIII digest of guide RNA expression vector:
pNTI661 …cgcggctgggaacgaaactctgggagctgcgattgg/GATCCTCGAAGCT/ttttagagctagaaatagcaagttaaaataaggctag…

digest  …cgcggctgggaacgaaactctgggagctgcgattgg    GATCCTCGAAGCT     ttttagagctagaaatagcaagttaaaataaggctag…

Gibson assembly of guide pool into expression vector:
digest  …cgcggctgggaacgaaactctgggagctgcgattgg                              ttttagagctagaaatagcaagttaaaataaggctag…
            |||||||||||||||||||||||||||||||||                              ||||||||||||||||||||||||||||||||||
PCR      5'-ggctgggaacgaaactctgggagctgcgattggcaCATTTCATTACCCGCAGAGCgttttagagctagaaatagcaagttaaaataaggc-3'

Gibson  …cgcggctgggaacgaaactctgggagctgcgattggcaCATTTCATTACCCGCAGAGCgttttagagctagaaatagcaagttaaaataaggctag…
```

Amplifying the barcode-to-guide assignment library (guide / R2 side)

```
Barcode-to-guide assignment primer:
NI-1038 5'-GTGACTGGAGTTCAGACGTGTGCTCTTCCGATCTcgaaactctgggagctgc
              TruSeq Read #2 primer

                     …cgcggctgggaacgaaactctgggagctgcgattggcaCATTTCATTACCCGCAGAGCgtttt…
                                  cgaaactctgggagctgc->
GTGACTGGAGTTCAGACGTGTGCTCTTCCGATCT
```

Figure S1. Guide RNA cloning strategy. The guide RNA expression vector is linearized by digestion with BamHI and HindIII. The guide RNA oligonucleotide pool is amplified by PCR with NM636 and NM637 primers to create a substrate for Gibson assembly. The assembly reaction reconstitutes an intact *P(RPR1)-sgRNA* expression cassette. PCR using NI-1038 will prime in the *P(RPR1)* region and amplify a fragment that includes the variable guide RNA sequence flanked by a portion of one Illumina sequencing adapter, corresponding to the P7 side of the library with the Read #2 primer site.

Cloning barcodes into pNTI661-guide library

```
Guide RNA expression vector:
pNTI661 …AGCTATCAGGCGCGCCACTTCACGCATGCTCAAGAGCTCGATCCGCAGGC…

Barcode amplification and assembly primers:
NI-1026 5'-CGCCACTTCACGCATGCNNNNNNNNNNNNNNNNNNNNNNNNNAGATCGGAAGAGCGTCGT
NI-1027 5'-TATCAGGCGCGCCACTTCACGCATGC
NI-1041 5'-TGCGGATCGAGCTCTTGAGCATGTAATACGACTCACTATAGCACGACGCTCTTCCGATCT

PCR amplification of barcoding insert:
NI-1027 TATCAGGCGCGCCACTTCACGCATGC
NI-1026          CGCCACTTCACGCATGCNNNNNNNNNNNNNNNNNNNNNNNNNAGATCGGAAGAGCGTCGT
NI-1041 reverse complement                                AGATCGGAAGAGCGTCGTGCTATAGTGAGTCGTATTACATGCTCAAGAGCTCGATCCGCA

PCR  5'-TATCAGGCGCGCCACTTCACGCATGCNNNNNNNNNNNNNNNNNNNNNNNNNAGATCGGAAGAGCGTCGTGCTATAGTGAGTCGTATTACATGCTCAAGAGCTCGATCCGCA-3'
                                        N25 barcode        <- TruSeq Read #1  <- P(T7 RNAP)

SphI digest of guide RNA expression vector (SphI leaves 3' overhangs):
pNTI661      …AGCTATCAGGCGCGCCACTTCACG/CATG/CTCAAGAGCTCGATCCGCAGGC…

pNTI661 …AGCTATCAGGCGCGCCACTTCACGCATG    CATGCTCAAGAGCTCGATCCGCAGGC…

Gibson assembly of barcoding fragment into expression vector:
vec …AGCTATCAGGCGCGCCACTTCACGCATG                                                               CATGCTCAAGAGCTCGATCCGCAGGC…
        |||||||||||||||||||||||||                                                               |||||||||||||||||||||||
PCR  5'-TATCAGGCGCGCCACTTCACGCATGCNNNNNNNNNNNNNNNNNNNNNNNNNAGATCGGAAGAGCGTCGTGCTATAGTGAGTCGTATTACATGCTCAAGAGCTCGATCCGCA-3'

pdt …AGCTATCAGGCGCGCCACTTCACGCATGCNNNNNNNNNNNNNNNNNNNNNNNNNAGATCGGAAGAGCGTCGTGCTATAGTGAGTCGTATTACATGCTCAAGAGCTCGATCCGCAGGC…
```

Amplifying the barcode-to-guide assignment library (barcode / R1 side)

```
Barcode-to-guide assignment primer:
NI-956 5'-AATGATACGGCGACCACCGAGATCTACACTCTTTCCCTACACGACGCTCTTCCGATC

    …AGCTATCAGGCGCGCCACTTCACGCATGCNNNNNNNNNNNNNNNNNNNNNNNNNAGATCGGAAGAGCGTCGTGCTATAGTGAGTCGTATTACATGCTCAAGAGCTCGATCCGCAGGC…
NI-956 reverse complement                                <-GATCGGAAGAGCGTCGTG
                                                                              TAGGGAAAGAGTGTAGATCTCGGTGGTCGCCGTATCATT
                                                                                                 <- Illumina P5
```

In vitro transcription and barcode counting library construction

```
In vitro transcription:
templ …AGCTATCAGGCGCGCCACTTCACGCATGCNNNNNNNNNNNNNNNNNNNNNNNNNAGATCGGAAGAGCGTCGTGCTATAGTGAGTCGTATTACATGCTCAAGAGCTCGATCCGCAGGC…
product rev compl       …TTCACGCATGCNNNNNNNNNNNNNNNNNNNNNNNNNAGATCGGAAGAGCGTCGTGC

Reverse transcription:
NI-1032 5'-GTGACTGGAGTTCAGACGTGTGCTCTTCCGATCTTATGCGGCCGGTACCCAG

GCACGACGCTCTTCCGATCTNNNNNNNNNNNNNNNNNNNNNNNNNGCATGCGTGAAGTGGCGCGCCTGATAGCTCGTTTAAACTGGGTACCGGCCGCATAGCGAACGTGTAGGGCAGCGTTTCC…
NI-1032 reverse complement                                                      <-CTGGGTACCGGCCGCATA
                                                                                                    AGATCGGAAGAGCACACGTCTGAA…
                                                                                                     TruSeq Read #2 / P7

PCR for barcode counting library:
i5         AAT…CGAGATCTACAC(i5)ACACTCTTTCCCTACACGACGCTCTTCCGATCT
RT product reverse complement                CACGACGCTCTTCCGATCTNN…NNGCA…ATAAGATCGGAAGAGCACACGTCTGAACTCCAGTCAC
i7 reverse complement                                                       AGATCGGAAGAGCACACGTCTGAACTCCAGTCAC(i7)ATC…TTG
```

Figure S2. Barcode cloning strategy. The guide RNA expression vector is linearized by digestion with SphI, an enzyme that leaves 3' overhangs that are not resected during Gibson assembly. The barcode insert is amplified by PCR from one oligonucleotide, NI-1026, containing a random N_{25} sequence, with flanking primers NI-1027 and NI-1041 that add flanking regions suitable for cloning. After assembly into the SphI site, the barcode is flanked immediately by a fragment of the Illumina TruSeq Read #1 site, with a T7 RNA polymerase promoter located just beyond this primer site. PCR using NI-956 will prime on this TruSeq Read #1 sequence and amplify a fragment that includes the barcode flanked by an Illumina sequencing adapter, corresponding to the P5 side of the library. In vitro transcription produces an RNA that includes the Read #1 site. Reverse transcription with a specific primer containing the TruSeq Read #2 / P7 sequence yields a cDNA that can be amplified into an Illumina library that includes the barcode.
