# Supplementary material for: A genome-scale CRISPR interference guide library enables comprehensive phenotypic profiling in yeast
Source: BMC Genomics. 2021 Mar 23;22:205. doi: 10.1186/s12864-021-07518-0 (PMC7986282; doi:10.1186/s12864-021-07518-0)
Supplement: Supplementary file 7 — Additional file 7: Figure S3. Receiver operating characteristic for logistic regression models of guide activity. [file 12864_2021_7518_MOESM7_ESM.pdf]

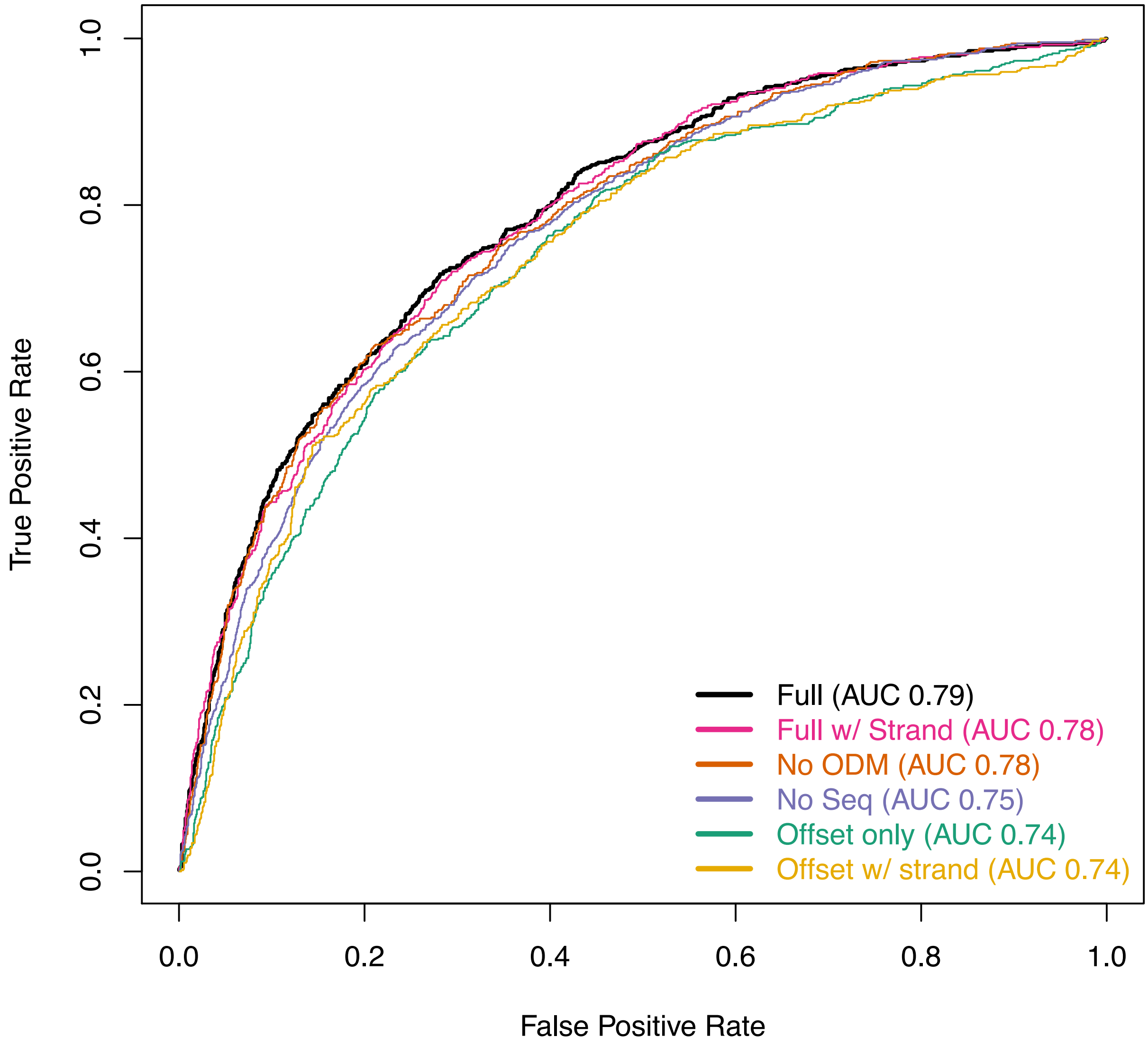

Figure S3. Accurate predictions of guide activity. Receiver operating characteristic for logistic regression models of guide activity. The full model includes methylation-based accessibility data (ODM-Seq) and guide sequence along with the position of the guide relative to the transcription start site. The "No ODM" and "No Seq" models exclude ODM-Seq and guide sequence parameters, respectively, and "Offset only" excludes both. The models "w/ Strand" include separate local regressions for guide activity based on the strand of the guide site relative to the target gene. All AUC values are calculated in the same 10-fold cross validation analysis.
