# Supplementary material for: A genome-scale CRISPR interference guide library enables comprehensive phenotypic profiling in yeast
Source: BMC Genomics. 2021 Mar 23;22:205. doi: 10.1186/s12864-021-07518-0 (PMC7986282; doi:10.1186/s12864-021-07518-0)
Supplement: Supplementary file 8 — Additional file 8: Table S8. Table of custom oligonucleotide sequences used in this study. [file 12864_2021_7518_MOESM8_ESM.pdf]

Name	Purpose	Sequence
**NM721**	subcloning dCas9-Mxi / TetR expression cassette	TTCCGTTGGTAGATACGTTGTTGACACTTCTAAATAAGCGCATAGCTTCAAAATGTTTCT
**NM734**	subcloning dCas9-Mxi / TetR expression cassette	TTTTAAAACCTAAGAGTCACTTTAAAATTTGCAAATTAAAGCCTTCGAGC
**NM613**	Sanger sequencing of pNTI647	CCTCGATAAGGTGCTTTCTGC
**NM614**	Sanger sequencing of pNTI647	CCAGATTCGCGTGGATGACT
**NM616**	Sanger sequencing of pNTI647	GGCGTACCATGAAAAGTACCC
**NM617**	Sanger sequencing of pNTI647	CCCCAAGAAATACGGCGGAT
**NM619**	Sanger sequencing of pNTI647	GAACTGGGGTCCCAAATCCT
**NM620**	Sanger sequencing of pNTI647	GCTGGTCTCAGATTTCAGAAAGG
**NM621**	Sanger sequencing of pNTI647	GGTCTTCAATTTCTCAAGTTTCAGTT
**NM622**	Sanger sequencing of pNTI647	TGTCCTCACCCTTACGTTGT
**NM623**	Sanger sequencing of pNTI647	ACCTGTCAGACGCCATTCTG
**NM726**	Sanger sequencing of pNTI647	CAGACGACAGATCTAAATGAC
**NM727**	Sanger sequencing of pNTI647	GCCATACATTAAAAGTTGAG
**NM728**	Sanger sequencing of pNTI647	CCGTGTACCTAAATGTACTTT
**NM729**	Sanger sequencing of pNTI647	AAGATCAAGAGCATCAAGTC
**p698Fwd**	subcloning *HIS3-MET17-URA3* cassette	TATTAGAAGAATATCCTGATATGCGGCATCAGAGCAGATT
**p698Rev**	subcloning *HIS3-MET17-URA3* cassette	CAACAATATTTTCACCTGAACATTTTTAATAAGGCAATAATATTAGGTATGT
**NI-993**	subcloning to replace *URA3* with *KlacLEU2*	GAAGGCTTTAATTTGCGGCCGGTACCCAGTTTAAACGAGCTCGATC
**NI-994**	subcloning to replace *URA3* with *KlacLEU2*	CGGTGATGACGGTGAAAACCGAATTCACCATTCAAATTGAACAG
**NI-995**	subcloning to replace *URA3* with *KlacLEU2*	TCTGTTCAATTTGAATGGTGAATTCGGTTTTCACCGTCATC
**NI-996**	subcloning to replace *URA3* with *KlacLEU2*	TTTTAATGTGTGCCGAACC
**KS524**	subcloning to remove dCas9-Mxi and TetR	CTCGTTTAAACTGGGTACCGGCCGCATAGCGAACGTGTAGGGCAG
**KS525**	subcloning to remove dCas9-Mxi and TetR	CTGCCCTACACGTTCGCTATGCGGCCGGTACCCAGTTTAAACGAG
**NI-1019**	subcloning to add barcoding site	TATACTAGTAATATGGTTCGGCACAC
**NI-1020**	subcloning to add barcoding site	ATCGAGCTCGCATGCAGTGGCGCGCCAGCTCGTTTAAACTGGGTAC
**NI-1030**	replacing gRNA cloning site	ATCAGGCGCGCCACTTCACGCATGCTCAAGAGCTCGATCCGCAGGCTAACCGGAA
**KS528**	Sanger sequencing of pNTI661	GTTCTTGGAAACGCTGCCCTAC
**KS529**	Sanger sequencing of pNTI661	GTTGCAATGCCAAGAAAGCAGC
**KS530**	Sanger sequencing of pNTI661	GAGGGAAGGGGAATATATTGTGCCTG
**KS531**	Sanger sequencing of pNTI661	GCACGTGATGAAAAGGACCCAG
**KS532**	Sanger sequencing of pNTI661	GTTATCTACACGACGGGGAGTCAG
**KS533**	Sanger sequencing of pNTI661	CCTTTTGCTGGCCTTTTGCTC
**NM636**	Guide amplification and cloning	GGCTGGGAACGAAACTCTGGGAGCTGCGATTGGCA
**NM637**	Guide amplification and cloning	GCCTTATTTTAACTTGCTATTTCTAGCTCTAAAAC
**NI-1026**	Barcode amplification and cloning	CGCCACTTCACGCATGCNNNNNNNNNNNNNNNNNNNNNNNNNAGATCGGAAGAGCGTCGT
**NI-1027**	Barcode amplification and cloning	TATCAGGCGCGCCACTTCACGCATGC
**NI-1041**	Barcode amplification and cloning	AGATCGGAAGAGCGTCGTGCTATAGTGAGTCGTATTACATGCTCAAGAGCTCGATCCGCA
**NI-956**	Barcode-to-guide assignment library PCR	GATCGGAAGAGCGTCGTGTAGGGAAAGAGTGTAGATCTCGGTGGTCGCCGTATCATT
**NI-1038**	Barcode-to-guide assignment library PCR	GTGACTGGAGTTCAGACGTGTGCTCTTCCGATCTCGAAACTCTGGGAGCTGC
**NI-1032**	Barcode reverse transcription	GTGACTGGAGTTCAGACGTGTGCTCTTCCGATCTTATGCGGCCGGTACCCAG

Table S8. Oligonucleotide sequences used in this study.
